# Intracranial hypotension headache complicated by retroclival subdural hematoma: clinical insights and literature review

**DOI:** 10.3389/fnins.2025.1754178

**Published:** 2025-12-18

**Authors:** Yi Yang, Dan Zhang, Qiaowei Zhang, Xingyue Hu, Wei Wang, Jin Wang

**Affiliations:** 1 Department of Neurology, Headache Center, Sir Run Run Shaw Hospital, School of Medicine, Zhejiang University, Hangzhou, China; 2 Department of Radiology, Sir Run Run Shaw Hospital, School of Medicine, Zhejiang University, Hangzhou, Zhejiang, China

**Keywords:** epidural blood patch (EBP), orthostatic headache, retroclival subdural hematoma (rcSDH), spontaneous intracranial hypotension (SIH), venous rupture

## Abstract

**Background:**

Retroclival subdural hematoma (rcSDH) secondary to spontaneous intracranial hypotension (SIH) is an exceedingly rare clinical entity, characterized by complex and incompletely understood pathophysiological mechanisms.

**Case:**

A 24-year-old female presented with acute and persistent orthostatic headache, with no history of trauma or anticoagulant therapy. Neuroimaging revealed subdural hematomas (SDH) located in the retroclival, infratentorial, and right frontal regions. It was hypothesized that veinous rupture, resulting from venous traction due to decreased cerebrospinal fluid (CSF) pressure, was the underlying mechanism. Following epidural blood patch (EBP) therapy, the patient exhibited marked symptomatic improvement and radiological resolution of hematomas on follow-up imaging.

**Conclusion:**

RcSDH is considered an uncommon complication of SIH, potentially resulting from venous rupture in the retroclival subdural space due to reduced CSF pressure. SIH should be considered in cases of rcSDH. The treatment is typically focused on addressing the underlying etiology, with early diagnosis and timely intervention being essential for achieving favorable outcomes. In cases of severe brainstem compression, hematoma evacuation should be performed in conjunction with EBP.

## 1 Introduction

Spontaneous intracranial hypotension (SIH) is a disabling disorder characterized by orthostatic headache secondary to spontaneous spinal cerebrospinal fluid (CSF) leaks and/or CSF hypotension (D'Antona et al., 2021; Dobrocky et al., 2022). In SIH, subdural fluid collections occur in approximately 50% of cases, with 60% presenting as hygromas and 40% as subacute to chronic hematomas (Schievink et al., 2005). The mechanism underlying subdural hematoma (SDH) primarily involves the expansion of the potential subdural-subarachnoid space, which facilitates the accumulation of blood or protein-rich fluid within this space. Furthermore, the phenomenon of brain sagging exerts downward traction on the bridging veins which connect the cortical veins to the dural sinuses, thereby increasing the risk of vein rupture and subsequent hemorrhage (Chen et al., 2016). The majority of these SDH or subdural fluid collections are bilateral, thin, and typically located over the cerebral convexities (Schievink et al., 2005; Schievink, 2006). However, it is noteworthy that subdural hematomas in the posterior fossa, particularly the retroclival subdural hematoma (rcSDH), which refers to SDH above the clivus, are relatively rare in SIH. Owing to its location outside the typical distribution of bridging veins, the pathogenesis of SDH in this region may differ from that of conventional SDH. The clivus is anatomically deep and in proximity to the brainstem and critical neurovascular structures. Therefore, subdural fluid collections or hematoma in this region, despite potentially being minor in volume, may carry substantial clinical implications and present greater challenges for radiological detection.

In light of the rarity and potentially unique characteristics of rcSDH associated with SIH, we present a detailed case of SIH with rcSDH, accompanied by a review of pertinent literature. This integrated approach seeks to improve clinical recognition of this uncommon complication.

## 2 Case report

A 24-year-old right-handed female presented to our hospital with a one-week history of orthostatic headaches. The headache, characterized by a sensation of fullness across the entire head (Visual Analogue Scale [VAS] score: 4/10), occurred immediately upon sitting or standing and was accompanied by neck and shoulder stiffness, while resolved immediately when the patient lay flat. She denied symptoms such as double vision, blurred vision, nausea, vomiting, or tinnitus. Upon admission, vital signs were stable: BP 102/60 mmHg, HR 57 bpm (regular), RR 20/min (unlabored), tympanic temperature 36.6 °C. The patient reported no pain (VAS 0/10) at rest in the supine position. No obvious focal neurological signs were observed, and meningeal signs were negative.

**Figure 1:**
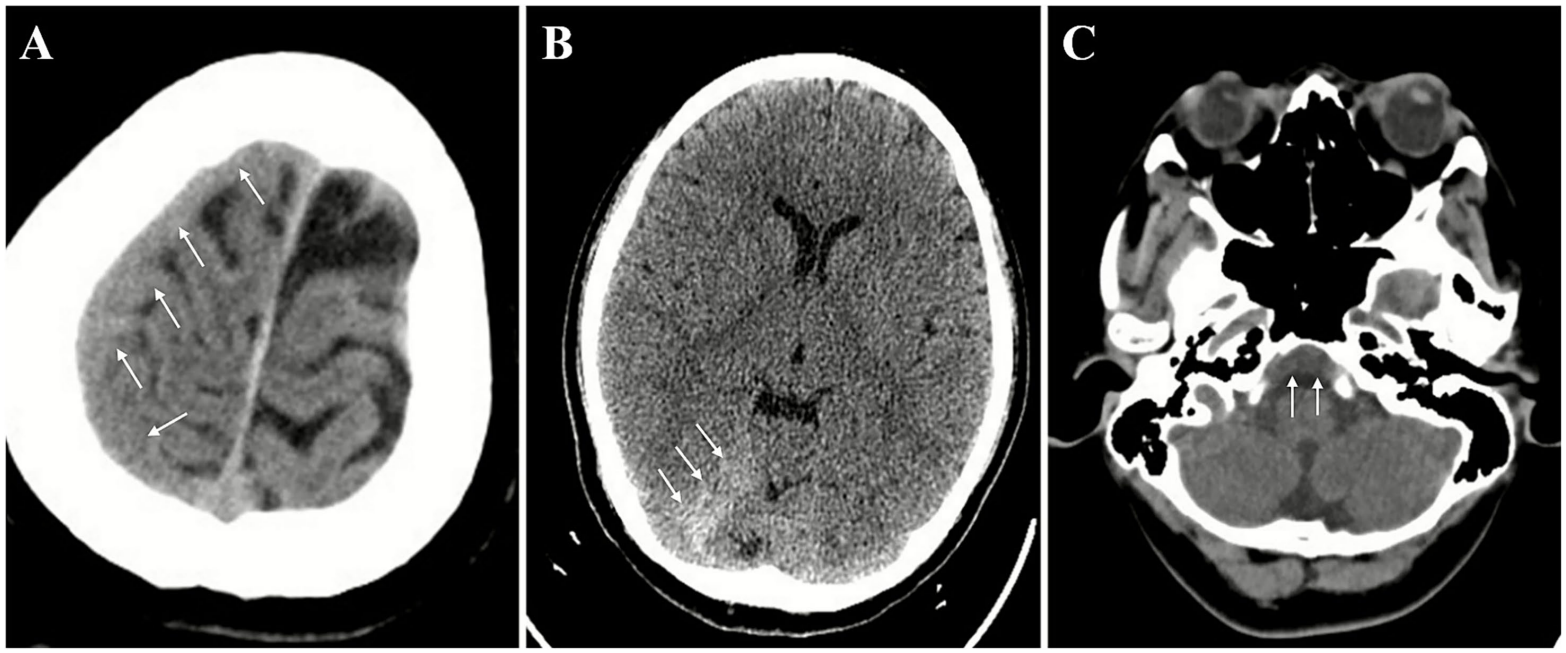
Initial cranial CT imaging upon admission. Subdural hematomas (arrows) were indicated in the right frontal parietal lobe **(A)**, right cerebellar tentorium **(B)**, and above the clivus **(C)**.

**Figure 2:**
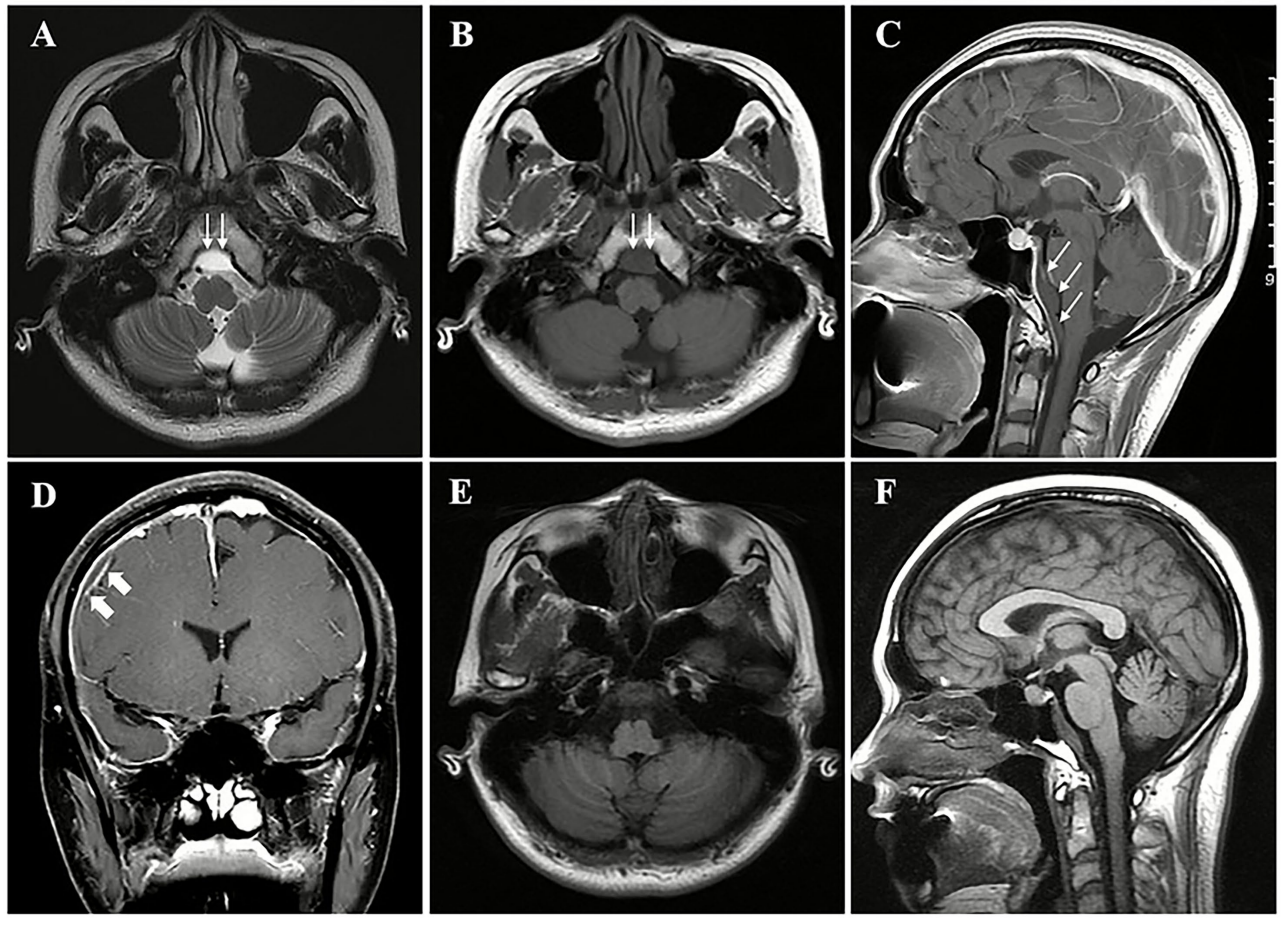
The cranial Magnetic Resonance Imaging (MRI) of the patient. A subdural hematoma (thin arrows) was observed in the region above the clivus with a hyperintense signal on T2-weighted imaging (T2WI) **(A)** and an isointense on T1-weighted imaging (T1WI) **(B)**. In the sagittal view, the subdural hematoma (thin arrows, maximum cross-sectional area 79.77 mm^2^) was evident in the region superior to the clivus and anterior to the brainstem, situated beneath the enhanced dura mater **(C)**. The diffuse, uniform enhancement of the dura mater, venous sinus enlargement, right parietal subdural hematoma (thick arrows) **(D)**, and pituitary fossa enlargement **(C)** were observed. Follow-up cranial MRI images **(E,F)** from 1 week after two epidural blood patches (EBP) demonstrated significant absorption of the subdural hematoma above the clivus.

The initial cranial CT scan revealed a right frontal parietal, right cerebellar tentorium, and a subdural hematoma above the clivus (see Figure 1). A cranial Magnetic Resonance Imaging (MRI) was performed, which revealed blood collection above the clivus in the subdural space beneath a uniformly enhanced dura mater. The collection appeared isointense on T1-weighted imaging (T1WI) and hyperintense on T2-weighted imaging (T2WI). The MRI also indicated uniform thickening and enhancement of the dura mater, venous sinus enlargement, and pituitary fossa expansion, consistent with the imaging features of spontaneous intracranial hypotension (SIH) with a Bern score of 9 (see Figures 2A–D).

**Figure 3:**
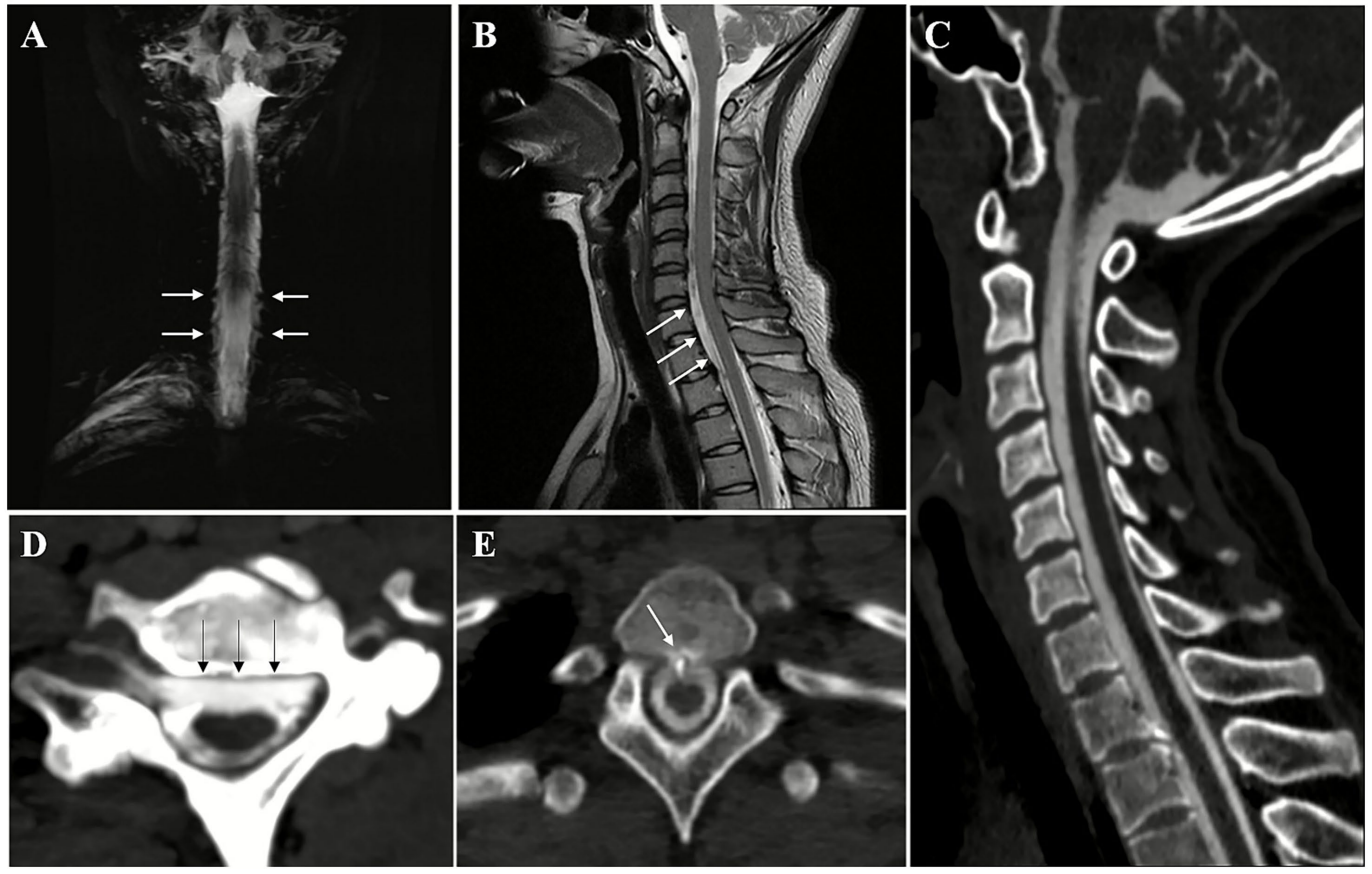
Evaluation of the patient’s spinal cerebrospinal fluid (CSF) leak. Heavily-T2 weighed MR myelography imaging showed increased CSF signal (white arrows) on the external side of the spine at the cervicothoracic junction **(A)**. Whole spine T2 sequence revealed epidural fluid collections (white arrows) in front of the spinal cord at the cervicothoracic junction **(B)**. Sagittal **(C)** and Axial **(D)** CT myelography (CTM) showed epidural fluid collections (black arrows) in front of the spinal cord at the cervicothoracic junction and upper thoracic segment. Osteophyte formation at the T1/2 vertebrae, projecting anteriorly toward the spinal cord, was observed, with the osteophyte (white arrows) almost touching the anterior side of the spinal cord, potentially piercing the dura **(E)**. The location of the CSF leak was suspected to be at the T1/2 osteophyte level.

Subsequent imaging with heavily T2-weighed MR myelography demonstrated CSF leak at the cervicothoracic junction (Figure 3). A T2-weighted whole spine MRI was performed, which revealed the presence of fluid accumulation in the anterior cervical and upper thoracic spine. CT myelography (CTM) revealed the presence of osteophyte formation at the lower cervical and upper thoracic vertebrae, with the T1-2 osteophytes potentially penetrating the dura. This indicated the potential location of the CSF leak may at the T1-2 osteophyte level.

The patient underwent two epidural blood patch (EBP) treatments. For the first time, the injection site was located at the T2-3 vertebral level, and an epidural catheter was inserted cephalad by 2–3 cm. A total of 13 mL autologous venous blood was injected cephalad, while an additional 5 mL was injected under pressure through the distal catheter. Then, 7 mL was injected caudally. One week later, a second EBP was performed at the T3-4 vertebral level with an epidural catheter inserted cephalad by 2–3 cm. A total of 11 mL was injected cephalad, while an additional 8 mL was injected under pressure through the distal catheter. Then, 6 mL was injected caudally. The patient’s orthostatic headaches significantly alleviated (VAS 1/10) after EBPs. One week following the second EBP, a follow-up head MRI demonstrated substantial improvement of the rcSDH (Figures 2E,F), with only a minimal residual right frontal–parietal subdural hematoma, corresponding to a Bern score of 1.

## 3 Discussion and literature review

This case report describes a rare presentation of SIH complicated by rcSDH and infratentorial SDH. Although SIH is well documented for its potential to induce SDH, it is extremely rare to be involved in the retroclival region and its underlying mechanisms remain poorly understood.

The pathophysiology of SIH frequently involves the presence of subdural hygromas and SDH, which may coexist and transition between forms (D'Antona et al., 2021; Dobrocky et al., 2022). According to the Monro-Kellie doctrine (Mokri, 2001), a reduction in CSF volume leads to an increase in blood volume within the cerebral veins, resulting in venous engorgement. This change, along with brain sagging, promotes fluid leakage into the potential subdural space, subsequently forming a hygroma. In the supratentorial and cerebellar convexity regions, this space is traversed by bridging veins. The significant traction on these engorged bridging veins makes them highly susceptible to rupture, leading to SDH (Schievink et al., 2005; Kristof et al., 2008; Dobrocky et al., 2019; Kim et al., 2019). Radiologically, hygromas typically manifest as homogeneous fluid signals without mass effect, while hematomas present with mixed signals and may exhibit layering and septation over time (Dobrocky et al., 2019).

**Figure 4:**
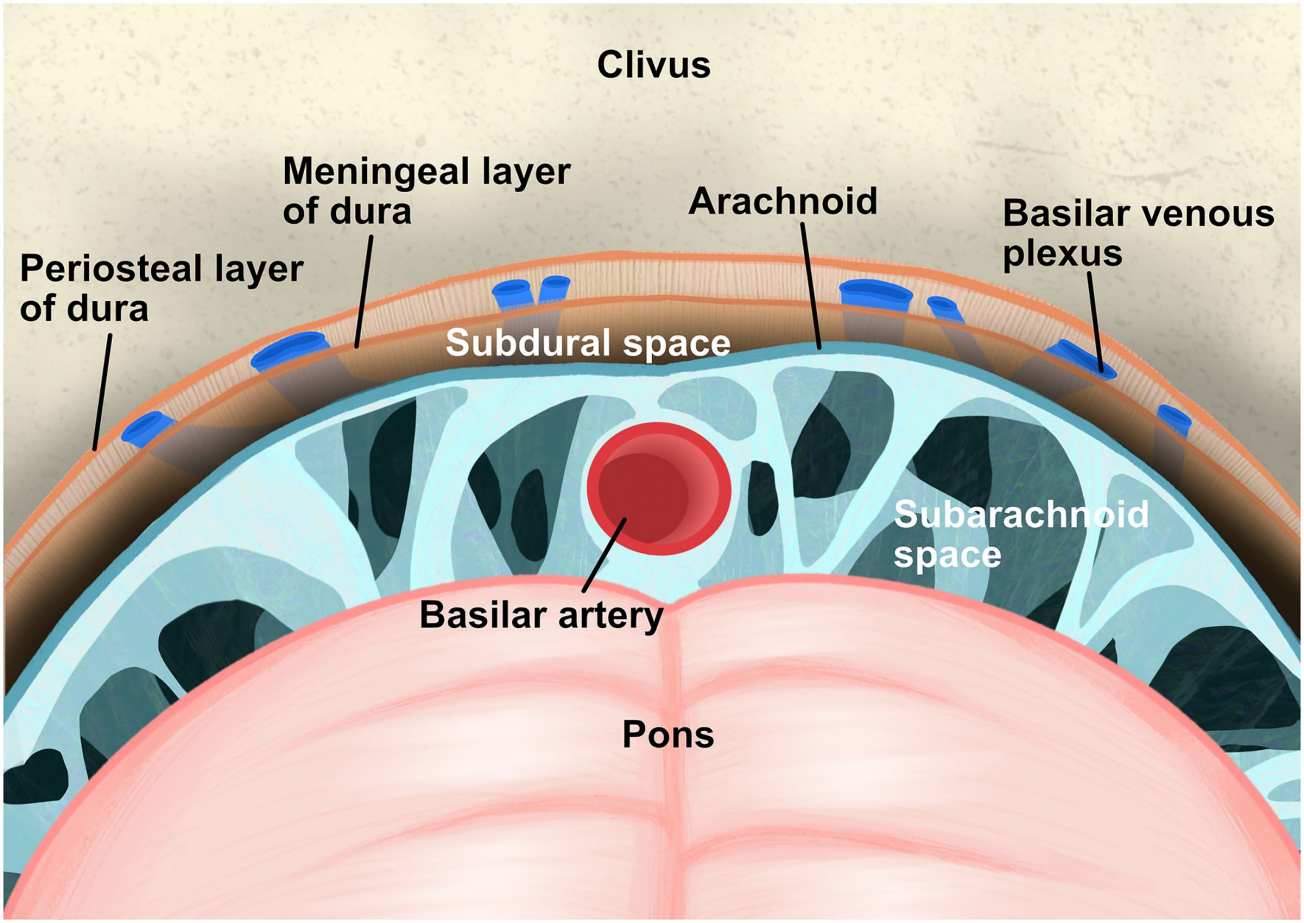
This schematic diagram depicts the retroclival subdural space. This anatomical compartment is a real, confined compartment, delineated anteriorly by the clival dura mater and posteriorly by the arachnoid mater. The basilar artery resides within the subarachnoid space, while the basilar venous plexus is situated in an interdural space between the periosteal and meningeal layers of the clival dura, both of which are separated from the subdural space.

The infrequency of retroclival subdural hematoma (rcSDH) can be attributed to its distinct anatomical structure (Krishnan et al., 2013). In contrast to the supratentorial subdual space, which is a large “potential” space, the retroclival subdural space is a “real,” confined compartment. It is bounded anteriorly by the clival dura and posteriorly by a relatively dense arachnoid layer (Figure 4) (Ayberk et al., 2011; Villanueva et al., 2023), thereby restricting the volume of blood that can accumulate and prevents the expansive growth typical of convexity subdural hematomas. The limited vascularization of this space further restricts hemorrhage. The basilar venous plexus is situated in an interdural space between the periosteal and meningeal layers of the clival dura, rather than in the subdural space (Caglar et al., 2020). Additionally, the basilar artery is safeguarded within the subarachnoid space by multiple membranes (e.g., anterior pontine membrane, medial pontomedullary membrane, Liliequist membrane) (Ayberk et al., 2011; Morris et al., 2022). Furthermore, any minor hemorrhage that does occur is likely to be rapidly redistributed into the spinal subdural space, further contributing to its rarity (Krishnan et al., 2013).

With regard to the relatively rare occurrence of rcSDH, literature review presented its etiology can be divided into two categories: traumatic and spontaneous (Corazzelli et al., 2024). The spectrum of spontaneous causes is diverse (see Table 1), including pituitary apoplexy (11/31 cases), anticoagulant therapy (7/31 cases, 1 combined with lumbar puncture), rupture of the posterior communicating artery aneurysm (3/31 cases) and infraclinoidal carotid aneurysm (1/31 cases), thrombocytopenia (1/31 cases), severe refractory hypertension (1/31 cases). The precise mechanism remains to be elucidated. It is hypothesized that rcSDH in pituitary apoplexy may result from direct extension from the sella (Azizyan et al., 2015), while anticoagulation or coagulopathy may elevate bleeding risk (Sever et al., 2017). SIH-induced rcSDH is very rare in previous reports. Notably, an illustrative case involved a 45-year-old female patient who, while on long-term oral contraceptives, developed rcSDH and a left frontal–parietal SDH following a lumbar puncture (Zelante et al., 2017). It is hypothesized that the pathogenesis of this condition may shares similarities with the mechanism observed in the SIH case. In this case, the young female patient diagnosed with SIH, who has no significant medication history or coagulopathy, presents with rcSDH primarily attributable to SIH. The proposed mechanism involves reduced CSF pressure in the posterior fossa, resulting in venous structure engorgement and traction, which subsequently leads to subdural venous tearing in the retroclival region and the formation of a hematoma.

**Table 1:** Summary of cases described in literature of spontaneous retroclival subdural hematomas.

Authors	Case No.	Age/gender	Pre-disposing factors	Treatment	Outcome
Han et al. (2016)	1	48/M	Nill	Conservative	Good
Schievink et al. (2001)	2	49/F	Nill	Conservative	Good
Narvid et al. (2016)	3	58/M	Nill	Conservative	Good
Narvid et al. (2016)	4	64/F	Nill	Conservative	Good
Narvid et al. (2016)	6	67/M	Nill	Conservative	Good
Matsumoto et al. (2020)	7	73/M	Nill	Conservative	Good
Narvid et al. (2016)	5	64/M	HTN	Conservative	Good
Kim et al. (2012)	8	83/F	Rupture of PcomA aneurysm	Endovascular coiling embolization	Good
Nowicki et al. (2023)	9	82/F	Rupture of PcomA aneurysm	Endovascular coiling embolization	Good
Saway et al. (2022)	10	87/F	Rupture of PcomA aneurysm	Endovascular coiling embolization	Good
Brock et al. (2010)	11	42/F	Rupture of Infraclinoidal Carotid aneurysm	Aneurysm clipping	NA
Mohamed et al. (2013)	12	37/M	Pituitary apoplexy	Surgery for Pituitary apoplexy; conservative for the SDH	Improved
Azizyan et al. (2015)	13	23/F	Pituitary apoplexy	Conservative	NA
Azizyan et al. (2015)	14	57/M	Pituitary apoplexy	Surgery for Pituitary apoplexy; conservative for the SDH	NA
Azizyan et al. (2015)	15	42/M	Pituitary apoplexy	Conservative	NA
Azizyan et al. (2015)	16	53/M	Pituitary apoplexy	Surgery for Pituitary apoplexy; conservative for the SDH	NA
Azizyan et al. (2015)	17	81/M	Pituitary apoplexy	Surgery for Pituitary apoplexy; conservative for the SDH	NA
Azizyan et al. (2015)	18	37/M	Pituitary apoplexy	Surgery for Pituitary apoplexy; conservative for the SDH	NA
Azizyan et al. (2015)	19	82/F	Pituitary apoplexy	Surgery for Pituitary apoplexy; conservative for the SDH	NA
Azizyan et al. (2015)	20	67/M	Pituitary apoplexy	Surgery for Pituitary apoplexy; conservative for the SDH	NA
Azizyan et al. (2015)	21	55/M	Pituitary apoplexy	Surgery for Pituitary apoplexy; conservative for the SDH	NA
Azizyan et al. (2015)	22	70/M	Pituitary apoplexy	Surgery for Pituitary apoplexy; conservative for the SDH	NA
Krishnan et al. (2013)	23	59/M	Thrombocytopenia	Attempted correction of coagulopathy	Died
Guilloton et al. (2000)	24	78/F	Anticoagulant therapy	Conservative	Good
van Rijn et al. (2003)	25	72/M	Anticoagulant therapy	NA	NA
Amalnath (2018)	26	30/F	Anticoagulant therapy	Conservative	Good
Bahadir (2021)	27	62/F	Anticoagulant therapy	Conservative	Good
Sever et al. (2017)	28	72/F	Anticoagulant therapy	Conservative	Good
Güvercin et al. (2025)	29	74/F	Anticoagulant therapy	Conservative	Good
Zelante et al. (2017)	30	45/F	Lumbar puncture; Anticoagulant therapy	Conservative	Good
The present case	31	24/F	SIH	EBP for SIH, conservative for the subdural hematoma	Good

NA, not available; HTN, hypertension; PcomA, posterior communicating aneurysm; SDH, subdural hematoma; SIH, spontaneous intracranial hypotension; EBP, epidural blood patch.

Clinically, rcSDH may manifest with severe headache and symptoms indicative of brainstem and upper cervical spinal cord compression, such as impaired consciousness, dysphagia, diplopia, or quadriparesis (Narvid et al., 2016; Corazzelli et al., 2024). In certain cases, blood may be observed extending through the foramen magnum into the spinal subdural space, reaching caudally to the level of the C3 vertebra (Casey et al., 2009; Krishnan et al., 2013; Han et al., 2016; Narvid et al., 2016). In cases of minimal hemorrhage, the rapid diffusion of blood into the spinal canal may result in misdiagnosis on CT scans.

The majority of rcSDH cases are resolved through conservative management, primarily addressing the underlying etiology. Surgical intervention is reserved for instances of progressive neurological deterioration or life-threatening mass effect (Narvid et al., 2016). Poor prognostic indicators include hematoma extension below C2, thickness greater than 1 cm, and gradual neurological decline (Corazzelli et al., 2024). For SIH-related rcSDH, no standardized management protocol exists. Epidural blood patch (EBP) serves as the primary etiological treatment for SIH and may also resolve associated subdural hematomas (Schievink, 2006; Mehta et al., 2023). However, significant brainstem compression or acute hydrocephalus with neurological deficits such as impaired consciousness may necessitate surgical interventions such as posterior fossa decompression or hematoma evacuation in conjunction with EBP (de Noronha et al., 2003; Chen et al., 2016; Zelante et al., 2017).

In this case, significant bone spurs were observed at the T1-2 vertebral level, which had pierced the dura mater, with fluid accumulation in the surrounding epidural space. It is speculated that this may be a type Ia condition (Schievink et al., 2016), caused by dural rupture due to a bony spur. The level of the bone spur is presumed to be the site of the leak, but precise localization still requires dynamic CTM or digital subtraction myelography (DSM) (Schievink et al., 2016). Despite the presence of SDH, no brainstem or cerebellar symptoms were observed. Following EBP treatment, imaging confirmed complete hematoma absorption with a favorable recovery.

## 4 Conclusion

RcSDH is recognized as a rare complication of SIH and is hypothesized to result from venous rupture within the retroclival subdural space due to decreased CSF volume. It is advisable to include SIH in the differential diagnosis in cases presenting with RcSDH. Treatment typically focuses on addressing the underlying CSF leak, with early diagnosis and timely intervention being crucial for achieving favorable outcomes. In cases of severe brainstem compression, hematoma evacuation should be performed in conjunction with EBP.

## Data Availability

The raw data supporting the conclusions of this article will be made available by the authors, without undue reservation.
